# A comparative study to evaluate the efficacy of EGF, FGF-2, and 0.3% (w/v) ofloxacin drops on eardrum regeneration

**DOI:** 10.1097/MD.0000000000007654

**Published:** 2017-07-28

**Authors:** Zhengcai Lou, Zihan Lou

**Affiliations:** a Department of Otorhinolaryngology, The Affiliated Yiwu Hospital of Wenzhou Medical University, Yiwu, Zhejiang; b Department of Clinical Medicine, Xinxiang Medical University, Xinxiang, Henan, China.

**Keywords:** epidermal growth factor, fibroblast growth factor-2, ofloxacin, traumatic, tympanic membrane perforation

## Abstract

**Background:**

Traumatic tympanic membrane perforations (TMPs) tend to spontaneous healing, however, large TMPs usually fail to healing. Clinical and experimental studies had demonstrated that growth factors accelerated the healing of large TMPs. The aim of this study was to compare the effects of growth factors and 0.3% (w/v) ofloxacin drops n the healing of human large TMPs.

**Methods:**

A total of 184 human large traumatic TMPs were randomly assigned to receive epidermal growth factor (EGF) treatment, fibroblast growth factor-2 (FGF-2) treatment, 0.3% (w/v) ofloxacin drops treatment, and conservative observation (only).

**Results:**

A total of 180 patients were analyzed in this study at the 6-month follow-up. The closure rates of the perforations in the EGF, FGF-2, 0.3% (w/v) ofloxacin drops, and conservative observation groups were 91.11%, 93.18%, 95.65%, and 82.22%, respectively, the closure rates did not significantly differ among the groups (*P* = .165). Similarly, pairwise comparisons did not reveal any significant between-group differences (*P* > .0083). However, the difference of the mean closure time was significant among the 4 groups (*P* < .001), pairwise comparisons showed that closure time was significantly longer in the observational group than in the other 3 groups (*P* < .001). Nevertheless, no significant difference in mean closure time was evident between any 2 treated groups (*P* > .0083). The mean hearing gain after 6 months was 11.49 ± 5.88 dB for the EGF group, 10.89 ± 5.16 dB for the FGF-2 group, 10.54 ± 5.56 dB for the ofloxacin group, and 9.29 ± 5.36 dB for the observation group. Differences in hearing improvement rates among the 4 groups were not statistically significant (*P* = .283).

**Conclusion:**

Epidermal growth factor, FGF-2, and 0.3% (w/v) ofloxacin drops accelerated the closure of large TMPs compared with conservative treatment. Surprisingly, neither the closure rate nor closure time differed significantly among the 3 treated groups. Further experimental studies to demonstrate whether 0.3% (w/v) ofloxacin per se accelerates the healing of TMPs will be interesting in the future.

## 1 Introduction

Traumatic tympanic membrane perforation (TMP) is commonly encountered in the otology clinic, but no optimal treatment has yet been defined. In general, topical application of antibiotic-containing ear drops is contraindicated, as this may cause secondary middle-ear infections.^[1–3]^

The safety and effectiveness of growth factors (epidermal growth factor [EGF],^[4,5]^ fibroblast growth factor-2 [FGF-2],^[6,7]^ and hyaluronidase [HA]^[8,9]^) used as treatments have been extensively investigated, and these agents have recently become widely used to treat both chronic and traumatic TMPs. The mechanisms by which growth factors facilitate eardrum healing have been well studied. Growth factors trigger proliferation of epithelial cells and fibroblasts and promote revascularization, thereby accelerating TMP closure.^[4–9]^ Clinical and experimental studies have suggested that topical application of EGF-2 improves the closure rate of traumatic TMPs and shortens the closure time.^[7,10–12]^ FGF-2 also promotes healing of glucocorticoid-induced TMPs.^[13,14]^ A few studies have shown that topical FGF-2 application facilitates the closure of chronic TMPs.^[15,16]^ Kato and Jackler^[15]^ treated chronic TMPs by applying 25 μL amounts of an FGF-2 solution delivered in a Gelfoam pledget every other day, for 6 days, in an experimental group [the control group received phosphate buffer solution (PBS)]. The closure rates were 81% in the FGF-2 group and 41% in the control group. EGF stimulates keratinocyte division in vitro and epidermal regeneration in vivo. Santa Maria et al^[17]^ found that EGF played an important role in wound healing of the eardrum, facilitating keratinocyte proliferation and migration. Other studies have found that topical EGF accelerates the closure rates of acute TMPs of rats and chronic TMPs of chinchillas.^[5,18,19]^ A recent clinical study found that both EGF and FGF-2 accelerated the closure of large traumatic TMPs in humans; the healing outcomes afforded by the 2 growth factors did not differ significantly.^[20]^ However, prior clinical studies have focused principally on the healing outcomes afforded by single growth factors.^[4,10–12,20,21]^ There is a paucity of clinical randomized controlled trials comparing different growth factors and other agent solutions.

Ofloxacin 0.3% (w/v) drops contain a broad-spectrum quinolone antibiotic widely used in practice to treat acute and chronic otitis externa and suppurative otitis media. One experimental study found that ofloxacin drops accelerate the closure rate to a greater extent than either saline or dexamethasone.^[22]^ In another study, ofloxacin drops, oral amoxicillin, or ofloxacin drops plus oral amoxicillin were used to treat traumatic TMPs in patients with secondary infections; only ofloxacin drops-alone accelerated the closure time.^[23]^ The objective of our study was to compare the healing outcomes afforded by EGF, FGF-2, and 0.3% (w/v) ofloxacin drops on large human traumatic TMPs; control groups underwent only conservative observation.

## 2 Materials and methods

The study was reviewed and approved by the Institutional Ethics Review Board of the Wenzhou Medical College-Affiliated Yiwu Hospital, China. All work was performed in compliance with the Helsinki Declaration. Written informed consent was obtained from all participants.

Study subjects were recruited consecutively among patients diagnosed with traumatic TMPs when visiting the Department of Otorhinolaryngology, Head and Neck Surgery, at the Affiliated Yiwu Hospital of WenZhou Medical College, between January 1, 2014 and June 30, 2016. The inclusion criteria were: a traumatic dry TMP; age >16 years; injury caused by a slap or blow to the ear, a blast, participation in sport, or direct penetration; and perforation of at least 25% of the pars tensa.

The exclusion criteria were: middle-ear infection or severe vertigo at the time of the initial hospital visit; ossicular chain damage or hyperplasia of granulation tissue (as revealed endoscopically and via computed tomography of the temporal bone); an additional (in particular, craniocerebral) injury associated with an inability to walk independently; and patient relocation from the region served by our hospital. Age, sex, date of injury, cause of injury, size of the TMP, and the presence or absence of otorrhea were recorded at the time of each hospital visit. Each patient was endoscopically examined after cerumen and/or blood clots in the external auditory canal (EAC) were removed. The tympanic membrane was photographed at the same time using a digital video camera, and the perforation size was recorded using ImageJ software (NIH, Bethesda, MD) and expressed as a percentage of the entire tympanic membrane (TM) area.^[24,25]^ A large perforation was defined as a perforation extending over >25% of the entire TM.^[26,27]^ Pure-tone audiograms were obtained using a Genemed Synthesis Inc 10 audiometer in a quiet room, at the initial and final visits or at 6 months after traumatic injury or treatment. The 4-dimensional method was used at 0.5, 1, 2, and 4 kHz to obtain the pure-tone average.

The principal investigator, aided by a registered nurse, allocated patients to various treatments, using simple random sampling. Specifically, consecutive subjects who met the inclusion criteria and signed the consent form were assigned random numbers generated by the SPSS for Windows software package (version 19.0; SPSS, Inc., Chicago, IL) that allocated them to one of the observational, EGF, FGF-2, or ofloxacin drops groups.

## 3 Technical methods

### 3.1 Control (observation) group

No intervention was offered but all patients underwent regular follow-up.

### 3.2 Treated group

The EAC was cleaned with a cotton bud soaked in povidone-iodine solution. Ears with traumatic TMPs then received the following treatments: first, 0.1 to 0.15 mL (2–3 drops) of recombinant human EGF solution (rhEGF; 200 μg/20 g; Yi Sheng, Zhuhai City, China); second, 0.1 to 0.15 mL (2–3 drops) of recombinant bovine FGF-2 solution (21,000 IU/5 mL) (Yi Sheng, Zhuhai City, China); or third, 0.1 to 0.15 mL (2–3 drops) of 0.3% (w/v) ofloxacin (WanHe, ShenZhen City, China). The agent solution was applied topically to the tympanic membrane once daily to keep the eardrum moist. The edge of the perforation was not approximated and no scaffolding material was used.

### 3.3 Follow-up

All eardrops were applied once daily by patients at home. The first follow-up occurred 2 to 3 days following treatment commencement. We confirmed that all patients correctly self-applied their drops. Dosages were carefully adjusted to ensure that the eardrum surface remained moist, thus neither dry nor overly liquid. Any inappropriate patient technique was corrected. Thereafter, follow-up was scheduled weekly until complete closure of the perforation was evident or for 6 months, whichever came first. Patients were advised to reduce the number of ear drops, and to take oral amoxicillin (with or without application of ofloxacin drops) if purulent otorrhoea developed. The tympanic membrane was repeatedly examined endoscopically, and color photographs were taken at all follow-up visits by a clinician blinded to treatment data.

### 3.4 Statistical analyses

To calculate the sample size, the study was powered at 80% and a type I error of 5% (α = 0.05) was used. A 15% difference in closure rate between the 3 treatment groups and control group was expected, and the sample size ratio of the EGF group, FGF-2 group, FLOX group, and control group was 1:1:1:1. With these values used to calculate sample size, it was determined that 44 patients were needed in each group using PASS software (ver. 11.0, Inc., Chicago, IL) software. Assuming a loss of 5%, the number of patients needed for each group was 46, for a total of 184 patients.

All statistical analyses were performed with the aid of SPSS software (version 19.0 for Windows). We explored the normality of continuous variables using the Kolmogorov–Smirnov test; normality was considered present if the *P* value was >.05. Among-group differences were compared by one-way analysis of variance (ANOVA) when the data were normally distributed. Post hoc multiple comparisons among groups were performed using the Bonferroni least significant difference test for homoscedasticity, and Dunnett's T3 test for heteroscedasticity. Total differences were estimated using the Kruskal–Wallis one-way ANOVA test. Among-group differences were compared with the aid of the Mann–Whitney *U* test when the data were not normally distributed. The chi-squared test was used to compare certain categorical variables (closure and infection rates). A *P* value ≤.05/6 (.0083) indicated that the post hoc multiple comparison reflected a significant difference. Otherwise, a *P* value <.05 was taken to reflect significance.

## 4 Results

### 4.1 Demographic characteristics

**Figure 1:**
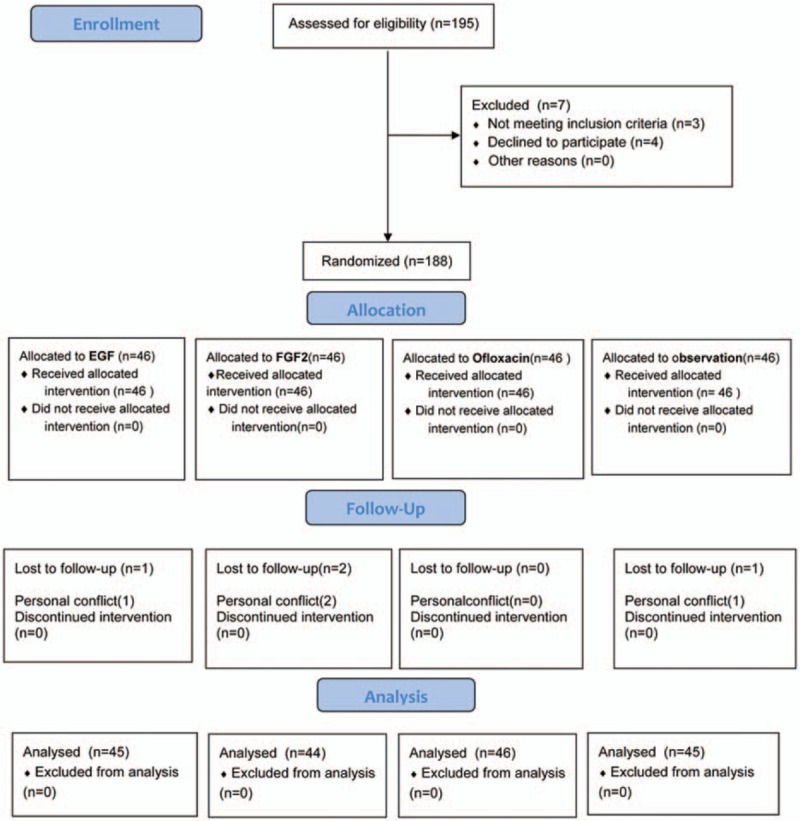
Enrollment, randomization, and follow-up of study participants.

In total, 184 patients met the exclusion criteria. Patients were randomly divided into 4 groups: EGF group (n = 46), FGF-2 group (n = 46), ofloxacin group (n = 46), and observation group (n = 46). Four patients were lost to follow-up (Fig. 1).

We ultimately included 180 patients (68 males, 112 females). The left ear was affected in 128 and the right ear in 52. Mean patient age was 36.7 ± 3.2 (range, 16–63) years. Demographic data are shown in Table 1. The mean ages of the EGF, FGF-2, ofloxacin drop, and observational groups were 35.96 ± 11.56, 37.68 ± 9.75, 35.09 ± 9.82, and 37.24 ± 11.00 years, respectively, and did not significantly differ among the 4 groups (*P* = .634). The comparisons of the differences in demographic data among the 4 groups included sex, age, side of the ear, perforation size (percent of tympanic membrane area), position of the perforation, duration, with or without closing to malleus, and hearing level. The result of normality tests of age, duration, perforation size, and hearing level among the 4 groups showed that age conformed with a normal distribution while the other 3 parameters were not normally distributed. No significant differences in patient sex, age, side of the ear, perforation size, position of perforation, duration, closing to malleus, or hearing level were observed among the 4 groups (*P* > .05), Pairwise comparisons showed that no significant differences in any of the demographic variables were evident between any 2 groups (*P* > .0083).

**Table 1:**
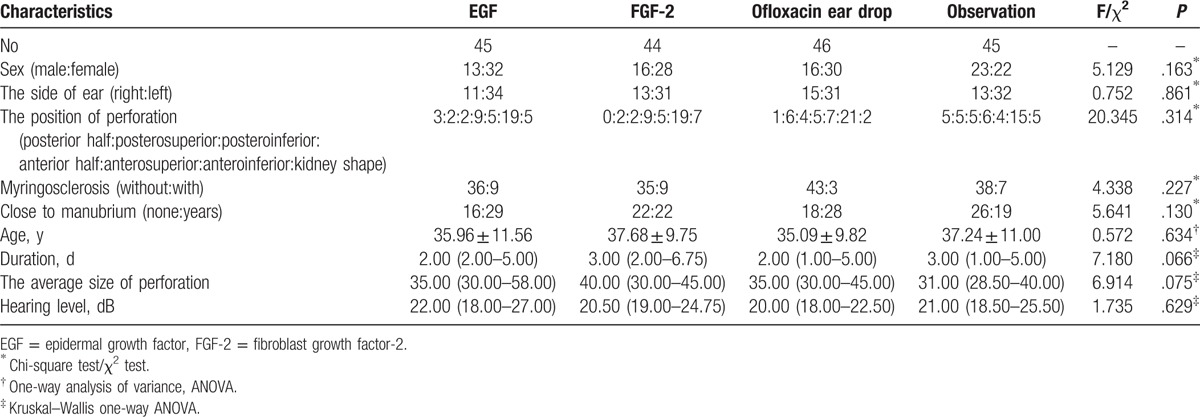
Demographic data of among groups.

A multivariate test of the association of closure time with confounding factors was performed using linear regression analysis. The results of the multiple linear regression analysis are shown in Table 2. A significant association between treatment class, whether close to the malleus, myringosclerosis, size of the perforation, and closure time was found. The healing time of perforations close to the malleus and without myringosclerosis was shortest. When the perforation was larger, the healing time was longer. The healing time was longest in the observation group. The correlation of other parameters and closure time was not significant (*P* > .05).

**Table 2:**
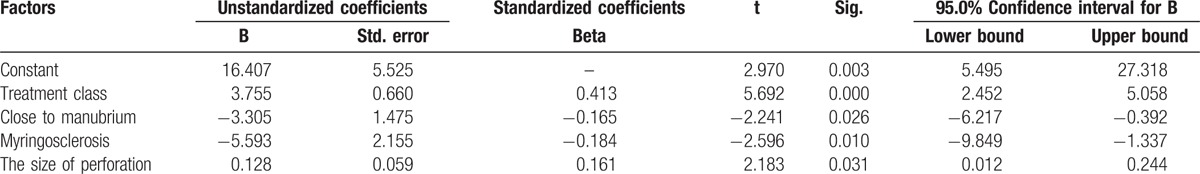
Multiple linear regression analysis.

### 4.2 Healing outcomes

**Figure 2:**
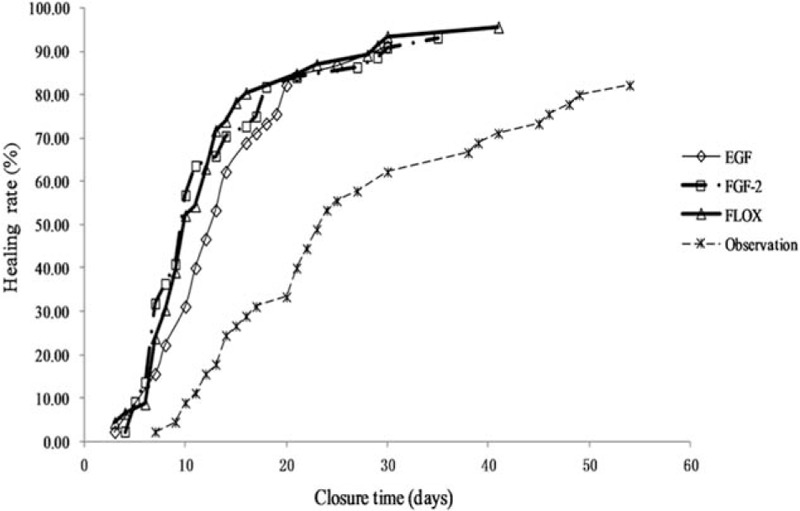
Time-to-closure rates of tympanic membrane perforations in the 4 groups.

The healing outcomes at 6 months are shown in Table 3 and Fig. 2. The closure rates did not differ significantly among the EGF, FGF-2, ofloxacin drops, and observational groups (91.11%, 93.18%, 95.65%, and 82.22%, respectively, *P* = .165). Pairwise comparisons showed that the closure rates did not differ significantly (EGF vs FGF-2, *P* = .716; EGF vs ofloxacin drops, *P* = .379; EGF vs observation, *P* = .215; FGF-2 vs ofloxacin drops, *P* = .608; FGF-2 vs observation, *P* = .116; and ofloxacin drops vs observation, *P* = .035).

**Table 3:**
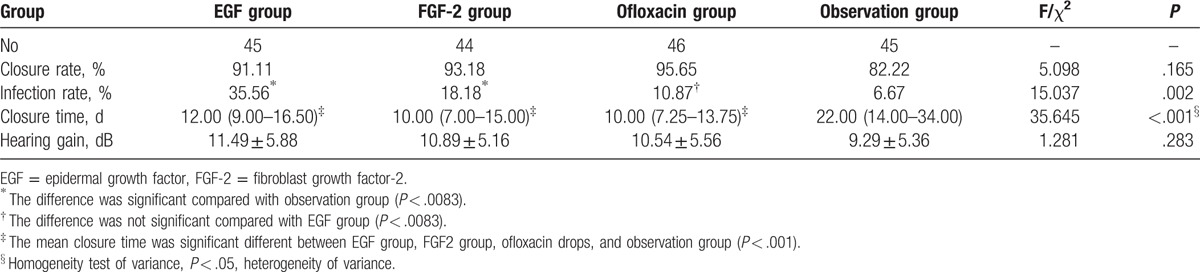
The healing outcome of EGF group, FGF-2 group, ofloxacin drops group, and observation group.

**Figure 3:**
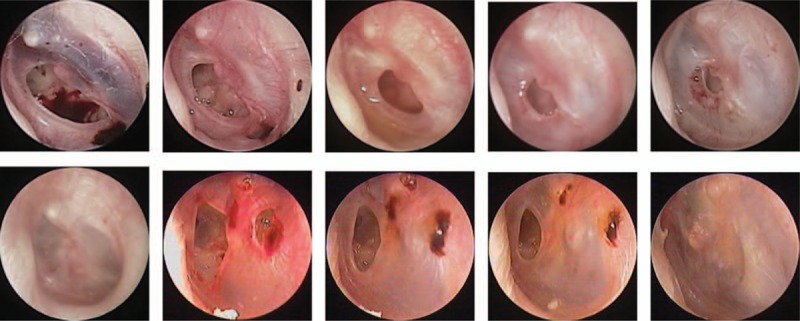
Extent of healing at various times after starting topical application of epidermal growth factor (EGF) and ofloxacin drops: (A) 3 days after perforation; (B–F) 3, 7, 10, 13, and 19 days after starting EGF treatment; (G) 1 day after perforation; (H–J) 6, 11, and 15 days after starting treatment with ofloxacin drops.

The mean closure time was 12.00 (9.00–16.50) days in the EGF group, 10.00 (7.00–15.00) days in the FGF-2 group, 10.00 (7.25–13.75) days in the ofloxacin drops group, and 22.00 (14.00–34.00) days in the observation group; the difference was significant (*P* < .001). Closure time was significantly longer in the observational than in the other 3 groups (*P* < .001). No significant difference in the mean closure time was evident between any 2 treatment groups (EGF vs FGF-2, *P* = .918; EGF vs ofloxacin drops, *P* = .986; and FGF-2 vs ofloxacin drops, *P* = 1.000). Figure 3 shows the healing process after EGF and ofloxacin drop treatment.

The mean hearing gain after 6 months was 11.49 ± 5.88 dB for the EGF group, 10.89 ± 5.16 dB for the FGF-2 group, 10.54 ± 5.56 dB for the ofloxacin group, and 9.29 ± 5.36 dB for the observation group. Differences in hearing improvement rates among the 4 groups were not statistically significant (*P* = .283). Pairwise comparisons revealed no significant differences in hearing gain between any 2 groups (EGF vs FGF-2, *P* = .606; EGF vs ofloxacin drops, *P* = .413; EGF vs observation, *P* = .059; FGF-2 vs ofloxacin drops, *P* = .768; FGF-2 vs observation, *P* = .172; and FLOX vs observation, *P* = .278).

### 4.3 Complications

During follow-up, some patients in all 3 treatment groups reported ear discomfort and otorrhoea, but no serious complications such as severe vertigo or external auditory meatus hyperkeratosis were observed.

Infection rates are shown in Table 3. The rate was 35.56% in the EGF group, 18.18% in the FGF-2 group, 10.87% in the ofloxacin drops group, and 6.67% in the observational group, and differed significantly among groups (*P* = .002). Pairwise comparisons revealed significant differences between the EGF and both the observational (*P* = .000), and ofloxacin drops groups (*P* = .005). No significant differences in infection rates were evident between the ofloxacin drops and observation groups (*P* = .477), the FGF-2 and observation groups (*P* = .099), the FGF-2 and ofloxacin drops groups (*P* = .324), or the EGF and FGF-2 groups (*P* = .065).

**Figure 4:**
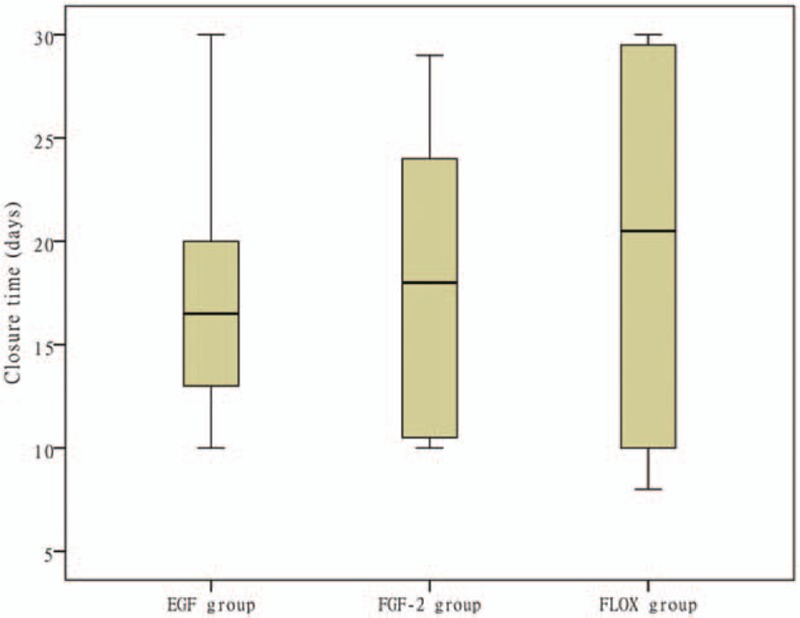
Closure times of infected ears in all groups. The central horizontal lines are the medians and the vertical lines in the closed boxes are the first and third quartiles.

Surprisingly, most of the infected ears in the 3 treatment groups achieved closure. The healing outcomes of the ear infections are shown in Table 4 and Fig. 4. The closure rates of infected ears were 87.5% in the EGF group, 87.5% in the FGF-2 group, and 80% in the ofloxacin drops group. Pairwise comparisons revealed significant differences in the closure rates of infected ears between the EGF and observation groups (*P* = .002) and the FGF-2 and observation groups (*P* = .004). No significant differences in closure rates were evident between the ofloxacin drops and observation groups (*P* = .014). The closure rates did not significantly differ between the 3 treatment groups (*P* > .0083). However, none of the infected ears achieved closure in the observation group. The mean closure times of infected ears did not differ significantly between the EGF, FGF-2, and ofloxacin drops groups (17.36 ± 5.46, 18.00 ± 8.04, and 19.75 ± 11.38 days, respectively; *P* = .846); the mean closure times of infected ears did not differ significantly between the 3 treatment groups (*P* > .0083).

**Table 4:**
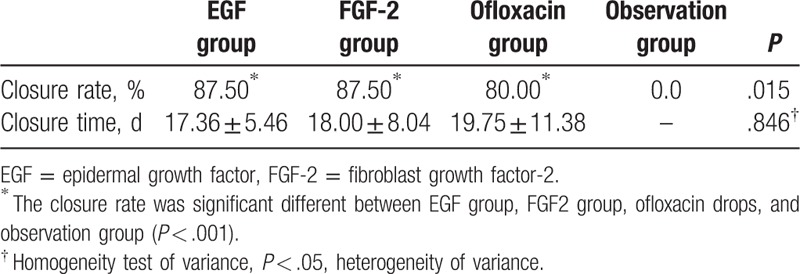
The healing outcome of EGF, FGF-2, ofloxacin ear drop, and observation group of infection ear.

Table 5 compares the closure rates and times between infected and noninfected ears in the same treatment groups. The closure rate did not significantly differ among the EGF (*P* = .535), FGF-2 (*P* = .513), and ofloxacin drops groups (*P* = .152). However, it was significantly less in the observation group (*P* = .001). Differences in closure time were significant between the observation group and EGF group (*P* = .002), but not between the observation group and the FGF-2 (*P* = .017) or ofloxacin drops groups (*P* = .047).

**Table 5:**
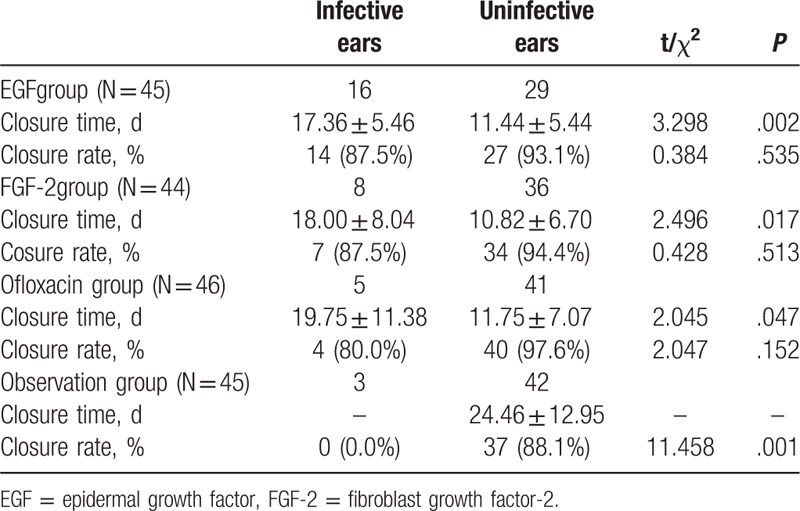
Comparison of closure rate and closure time between the infective and uninfective ears in the same treatment group.

**Figure 5:**
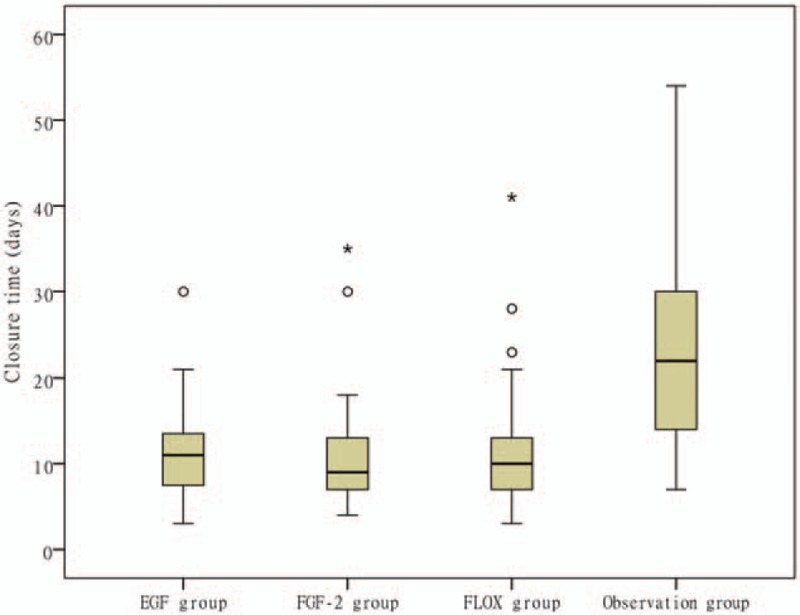
Mean closure times in each group (infected cases were eliminated). The central horizontal lines indicate the median values; the vertical lines in the closed box show the first and third of the 4 quartiles. ° and ^∗^indicate discrete and extremely abnormal values, respectively. The vertical values were 30 days in the epidermal growth factor (EGF) group; 30 and 35 days in the fibroblast growth factor-2 (FGF-2) group; and 23, 28, and 41 days in the ofloxacin drops group. In the EGF group, the median value was 12 days, the 25th percentile was 9 days, and the 75th percentile was 16.50 days. In the FGF-2 group, these values were 10, 7, and 15 days. In the ofloxacin drops group, the values were 10, 7.25, and 13.75 days. In the observational group, they were 22, 14, and 34 days. The English in this document has been checked by at least 2 professional editors, both native speakers of English. For a certificate, please see: http://www.textcheck.com/certificate/p3ntjq.

When infected cases were eliminated from statistical analyses, healing outcomes at 6 months were as shown in Table 6 and Fig. 5. The closure rates did not significantly differ among the EGF, FGF-2, ofloxacin drops, and observation groups (93.10%, 94.44%, 97.56%, and 88.10%, respectively, *P* = .372), as revealed by pairwise comparisons (EGF vs FGF-2, *P* = .824; EGF vs ofloxacin drops, *P* = .368; EGF vs observation, *P* = .478; FGF-2 vs ofloxacin drops, *P* = .479; FGF-2 vs observation, *P* = .319; and ofloxacin drops vs observation, *P* = .082).

**Table 6:**
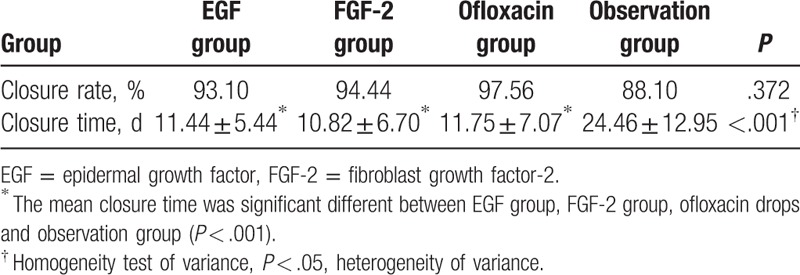
The healing outcome of EGF, FGF-2, ofloxacin drops, and observation groups after eliminating infection ear.

The mean closure times differed significantly among the EGF, FGF-2, ofloxacin drops, and observational groups (11.44 ± 5.44, 10.82 ± 6.70, 11.75 ± 7.07, and 24.46 ± 12.95 days, respectively; *P* < .001). Pairwise comparisons between groups showed that the mean closure time was significantly longer in the observational group compared to the other 3 groups (*P* < .001). However, no significant difference in mean closure time was evident between any 2 treatment groups (EGF vs FGF-2, *P* = .999; EGF vs ofloxacin drops, *P* = 1.000; FGF-2 vs ofloxacin drops, *P* = .993).

## 5 Discussion

Traumatic TMPs tend to heal spontaneously, but slowly. Delayed healing may cause long-term tinnitus, conductive hearing loss, chronic TMP problems, and severe diminishment of quality of life.^[1–3]^ In recent years, some growth factors (e.g., EGF, FGF-2, and HA) have been used to aid TMP healing.^[4–21]^ Of the many growth factors currently known, the most important in terms of eardrum healing are EGF and FGF-2.

The safety and effectiveness of EGF and bFGF used to treat TMPs have been well-demonstrated.^[4–21,28,29]^ This study also demonstrated that topical application of growth factors (EGF or FGF-2) significantly shortens the closure time compared to conservative treatment. In addition, we did not use scaffolding material nor did we approximate the perforation edges in our study. Each test group received only a single agent solution (EGF, FGF-2, or ofloxacin). Thus, some confounding factors were eliminated, and we considered that the promoting effects of traumatic TMP healing was mainly due to spontaneous healing of the eardrum and the biological effects exerted by these agents.

Theoretically, the healing outcome of the 2 growth factor groups was superior to that of the ofloxacin drops group. However, surprisingly, neither EGF nor FGF-2 significantly improved the closure rate or shortened the closure time relative to the ofloxacin drops group in this study. Most perforations closed completely within 1 to 2 weeks of the commencement of treatment in the 3 test groups. This unexpected finding raises certain questions: first, does the topical application of exogenous growth factors actually promote eardrum healing? Second, does ofloxacin act in a similar manner to growth factors? third, ofloxacin ear drops include a broad-spectrum quinolone antibiotic commonly used worldwide to treat acute, chronic, and suppurative otitis media in adults and children. Only an experimental study of ciprofloxacin/dexamethasone and ofloxacin on TMP healing showed ciprofloxacin/dexamethasone delays healing of experimental TMPs; however, isotonic sodium chloride solution control and ofloxacin-exposed TMPs healed at similar rates.^[23]^ There is no other evidence that ofloxacin accelerates eardrum repair per se.^[30–32]^ Is a moist eardrum environment vital for healing regardless of the topical application of agents in solution?

Two previous studies have shown that neither closure rate nor closure time differed significantly between a low dose of growth factors and a PBS group, and it was recommended that the growth factor doses should be increased.^[33,34]^ However, this may increase the risks of myringitis, otorrhoea, and other complications. In addition, long-term applications of high doses of growth factors may inhibit collagen synthesis, resulting in tympanic membrane reperforation and middle-ear cholesteatoma.^[33,35]^ It is important to note that an excessive middle-ear liquid environment may cause human ear discomfort and treatment interruptions or intermittent use of drops, thereby compromising healing. In this study, we constantly adjusted ear drops doses to ensure that the surface of the eardrum was moist, but not excessively dry or drenched.

Previous observations have suggested that moist TMPs healed more rapidly and completely than did dry wounds.^[36,37]^ Moisture prevents edge crust formation and outward epithelial migration. It also facilitates the actions of endogenous growth factors, cytokines, and chemokines, promoting epithelial cell and fibroblast growth and the establishment of a provisional wound matrix, accelerating, in turn, healing of the eardrum.^[36,37]^ We speculate that exogenous growth factors have minimal effects on eardrum healing; the moistness of the environment is the critical factor. Topical application of growth factors merely increases the local activities of endogenous factors, or remodels the biochemistry of spontaneous healing.^[38]^ Nevertheless, an excess liquid (wet) environment impairs healing and damages surrounding tissue, triggering peri-wound maceration.^[37]^ A wet environment is associated with an increased incidence of middle-ear cholesteatoma.^[36,39]^ In addition, a recent experimental study demonstrated that an acidic pH environment speeded up the healing of traumatic TMPs by suppressing infection and reducing catabolic and proteolytic activities, and/or providing ongoing epithelization at the edges of the perforation. However, an alkaline pH delayed eardrum healing.^[40]^ Ofloxacin ear drops constitute an acidic solution preparation.

None of our patients developed severe vertigo, external auditory meatus hyperkeratosis, or otomycosis. Some patients in the 3 test groups experienced ear discomfort and developed otorrhoea. The infection rates differed significantly among the 4 groups; the EGF and FGF-2 groups had higher rates of otorrhoea, and the infection rate in the ofloxacin drops group was lower. Perhaps because patients did not adequately control the numbers of ear drops, or because of individual differences in the structure of the EAC, the amounts of middle-ear liquid residue varied, triggering infections. However, ofloxacin has anti-inflammatory action. Kälicke et al^[41]^ found that topical growth factors enhanced infection of traumatic wounds. Surprisingly, infection did not inhibit TMP closure. A previous study found that secondary middle-ear infection did not compromise spontaneous healing of traumatic TMPs.^[23]^ Most infected ears of all groups closed completely upon prescription of oral amoxicillin; neither the closure rate nor closure time of infected ears differed significantly among the 3 treatment groups in this study. In the observational group, none of the infected perforations had closed, rendering statistical analysis impossible. Previous studies have suggested that FGF-2 and EGF could reverse the inhibition of contraction caused by bacteria and indeed facilitated the healing of wounds damaged by bacteria.^[42]^ Other studies have reported that acute middle-ear infection actually induced TMP healing.^[43]^

The major limitations of our study are the lack of a saline control group and the absence of histological data. However, it is difficult to histologically examine the human eardrum. The use of saline alone to treat human TMPs is ethically problematic.

## 6 Conclusion

Topical applications of EGF, FGF-2, and 0.3% (w/v) ofloxacin drops accelerated the closure of large human traumatic TMPs compared with conservative treatment. Surprisingly, neither the closure rate nor closure time differed significantly among the 3 treated groups. Topical application of 0.3% (w/v) ofloxacin drops aids in the healing of traumatic TMPs, and does not increase the rate of ear infections. Otolaryngologists should consider the drugs available, the economic circumstances of the patient, and the size of the perforation, to optimize treatment. In general, 0.3% (w/v) ofloxacin ear drops provide the most convenient treatment and should be considered in clinic for the treatment of traumatic TMPs. However, further experimental studies to demonstrate whether ofloxacin per se accelerates the healing of TMP will be interesting in the future.
